# Indicators of Mental Health in Various Iranian Populations

**DOI:** 10.5812/ircmj.14292

**Published:** 2014-02-05

**Authors:** Khosro Mohamadi, Khodabakhsh Ahmadi, Ali Fathi Ashtiani, Parviz Azad Fallah, Abbas Ebadi, Emad Yahaghi

**Affiliations:** 1 Behavioral Sciences Research Center, Baqiyatallah University of Medical Sciences, Tehran, IR Iran; 2 Baqiyatallah University of Medical Sciences,Tehran, IR Iran; 3 TarbiatModarres University, Tehran, IR Iran; 4 Young Researchers and Elite Club, North Tehran Branch, Islamic Azad University, Tehran, IR Iran

**Keywords:** Mental Health, Indicators and Reagents, Iran

## Abstract

**Background:**

Promoting mental health and preventing mental disorders are of the main concerns for every country. Achieving these goals requires effective indexes for evaluating mental health. Therefore, to develop mental health enhancement programs in Iran, there is a need to measure the state of mental health in Iran.

**Objectives:**

This study aimed to select a set of mental health indicators that can be used to monitor the status of mental health in Iran.

**Materials and Methods:**

This research work used Q-methodology which combines both quantitative and qualitative research methods for establishment of mental health indicators in Iran. In this study, 30 participants were chosen by purposive sampling from different types of professionals in the field of mental health.

**Results:**

Twenty seven mental health indicators were obtained from the Q-methodology. The most important indicators obtained in this study are as follows: annual prevalence of mental disorders, suicide rates, number of mental health professionals, mental health expenditures and suicide related deaths.

**Conclusions:**

This study provides mental health indices for measuring mental health status in Iran. These mental health indices can be used to measure progress in the reform policies and community mental health services.

## 1. Background

Mental disorders are prevalent across the world and are disabling and very costly (1, 2). Approximately 450 million people worldwide suffer from mental, neurological or behavioral disorders at any given time, and studies show that about one-fifth will experience a psychiatric disorder within a given year (2-4). In addition, the economic and social burdens of mental disorders are rising steadily, and according to the global burden of disease project of the World Health Organization (WHO), mental disorders such as depression, schizophrenia, bipolar affective disorder, and alcohol abuse are among the 20 leading causes of disability (5). Especially, major depression, was ranked the third in terms of contribution to total disease burden in 2004 and is expected to be the first in 2030 (1-5). Point prevalence of mental disorders in the Islamic Republic of Iran is estimated to be approximately 22 percent (6, 7). Iran's national mental health program (INMHP) was created in 1986 and was adopted by the Ministry of Health and Medical Education in 1988 (8). In 1989, the mental health and primary health care programs were integrated (8, 9). Through the integration of mental health care in general health care, a significant portion of the Iranian population is now covered by mental health services which are accessible, affordable and high quality (8, 10). Enhancement of mental health services in Iran since 1988 has been remarkable (8, 9). A comprehensive and integrated mental health legislation is still lacking; and especially there are few valid indices for monitoring and evaluating mental health programs. Valid indices of mental health are important in assessing and improving quality of mental health services as they can show variations in the quality of care (10, 11). Mental health indices can also be used as a catalyst to facilitate quality improvement initiatives in the mental health services (11). To reduce the social and economic impact of mental health problems, it is essential that the public status and the general level of mental health of the nation would be determined, the changes monitored periodically, and efforts to improve the situation would be undertaken, if deemed necessary. Especially, with the currently set priorities regarding availability of the data that will be used in policy making, it is necessary to create various mental health indices through surveys or statistical reports and use them in policy making programs (5). Mental health indices are necessary for monitoring people’s mental health status, developing mental health programs, and evaluating the performance of policies. In addition, mental health indices are essential for comparison among various countries (5, 12, 13). To determine whether mental health status in Iran is enhancing, it is necessary to measure and track progress. To achieve this goal, Behavioral Sciences Research Center of Baqiyatallah University of Medical Sciences is running a project with the aim of developing a sustainable core set of mental health indices for Iran. This project will certainly support the activities of INMHP. These indices will provide a measure for monitoring the mental health programs in Iran. Also, mental health Indices would help in determining overall status of the mental health in Iran.

## 2. Objectives

This study was conducted in Iran to establish and offer mental health indices that can be used in monitoring and determining the current level of mental health in Iran.

## 3. Materials and Methods

This study was performed based on the Q-methodology between October 2011 and March 2013 in Iran (Tehran). The Q-methodology includes both qualitative and quantitative procedures. In the Q-methodology, the strengths of both quantitative and qualitative research methods are included (14). Q-methodology combines strengths of a rigorous statistical analysis (factor analysis) and the benefits of qualitative research methods which are used for subjective studies (14, 15). Q-method has been used in various fields; for example, studies of health and illness (16) and exploration of emotions such as jealousy (17). The current study was based on six stages:

### 3.1. Development of Q Sample Statements

The original 246 Q set (A preliminary set of indices) were prepared by using experts' opinion, focus group discussions, interviews and journal articles covering a wider variety of views available on the mental health topics. The focus group discussions and interviews were conducted at a convenient time and place for the participants. The mean time of interviews was about 60 minutes. Focus group discussions and interviews were conducted until enough data was obtained. After 4 focus group discussions and 5 interviews, no extra information was obtainable. Finally, 98 statements were selected.

### 3.2. Selection of Participants for the Q Sort

In studies using Q-methodology, samples are carefully selected rather than randomized so that variability in a specific case or situation can be analyzed (14). Thirty participants (different professionals in the field of mental health) were selected by purposive sampling type of Homogeneous sampling for the Q study. According to some research studies, 30 participants would be sufficient for performing a Q study. With large number of participants, some issues might arise, such as removing some essential qualities present in the data (14, 18). Inclusion criteria were: 1- Minimum 20 years of professional or managerial experience in the field of mental health and health care, 2 - Minimum master's degree. All participants were assured about the confidentiality of their responses and anonymity of their participation and verbal informed consent was obtained. This study was approved in 2011 by the Ethics Committee of Baqiyatallah University of Medical Sciences (No. 90-303 Date: 27/7/2011).

### 3.3. Q-sorting

Q sort information package containing guidelines for the sorting procedure was provided and each participant received packet of guidelines for the sorting. The package contains the following items:

a- Q-Statements (98 indexes)

b- Ranking table of indices , ranked from one to nine Data Entry:

Data entry was done in two steps:

a- Sorting each participant's data, which was recorded using Qcom.exe program.

b- Determining the correlation between the participants' Q sorting which was performed using SPSS for windows, Version-15.

### 3.4. Statistical Analysis

Statistical analysis was done in two steps:

a- For the extraction of Q-sorts, the principal components analysis method was used.

b- Varimax with kaiser normalization method was used for the rotation of the extracted factors (14, 18).

### 3.5. Factor Interpretation

In this study, principal component analysis (PCA) was used to downsize the data set into a more manageable size while retaining as much of the original information as possible (18). Based on the factor score, Q-methodology can reveal the main shared viewpoints on a particular Subject but cannot provide information about the proportion of the population that such notions would apply to. Not with standing, the differences between two or more factor scores helps in identifying the extent of common ranking between various statements and also the degree of disagreement between the factors (4, 18). The qualitative procedure included the development of 98 Q statements from focus groups, expert opinion and interviews. Scaled questions (from mostlydisagree to mostly agree, graded one to nine) were used for the Q-sort. In this regard, consideration was given to factor score (18), consensus and divergent statements andtranscripts (qualitative data) from group discussions.

## 4. Results

Thirty experts participated in this study. Participants were between the ages of 35–60 with 25 being male and 5 female. They had at least 20 years of professional or managerial experience in the field of mental health and health care. Details of the participants’ gender and professional characteristics are shown in Table 1. 

**Table 1.** Four Professional Panels Involved in the Q-Methodology Process

Characteristics	No.	%
**Gender**		
Male	25	83.3
Female	5	16.7
**Profession**		
Psychiatrist	3	10
Psychologist	18	60
Medical doctor other than psychiatrist	4	13.3
Counselor	5	16.7
**Education**		
Professor	2	6.6
Assistant and Associate Professor	8	26.7
PhD student	12	40
MA	8	26.7

An initial set of indices (A total of 246 indices) was obtained from a number of foreign scientific resources. The experts commented and discussed this list of mental health indices during focus group discussions and interviews. Indices obtained from this phase were edited by the researchers. During this round (Q sorting Phase) the Q-methodology panelists were ranking indices on continuous integer 9-point scales for validity and importance with 1 = mostly disagree and 9 = mostly agree rating. Twenty-seven indices for which factor loading were higher (more than 0.7) were selected. To evaluate the reliability, sorting was repeated by 10 experts included in the study and an independent expert group. As experts re-confirmed the factor loading to be 73%, sorting was deemed acceptable.

### 4.1. Categories of Indices

Our study recommends 27 possible indices, which can be divided into 3 groups. Based on the results of the Factor Interpretation and Extraction of Q sorts using Principal Component Analysis on the suitability of index category, adequacy was high for the mental health system, mental health, and mental health factors among the 3 highest ranking domains. Among the sub domains, indices belonging to mental health system such as human resource, and indices belonging to mental health status such as mental health problems attained a high score; more specifically the results on the sub domain, ‘Finance’ (belonging to mental health system) and ‘Environmental Factors’ (belonging to mental health factor) showed high importance (Table 2). 

**Table 2.** Mental Health Indices Categories

Domain	Ranking
**Mental health status**	2
Positive mental health	7
Mental health problems	2
**Mental health factor**	3
Environmental factors	4
Personal factors	6
**Mental health system**	1
Mental health Service	5
Human resources	1
Finance	3

### 4.2. Mental Health Indices 

Our results proposed a total of 27 mental health indices. Of these, 19 indices were based on statistical information, and 8 indices required survey data. Among the 27 indices, 14 belonged to ‘mental health system’ which is suggestive of the importance of resources such as Mental Health Services , human resources and finance in understanding the current situation of mental health and making related policies in Iran. In addition, indices in the sub domain of ‘mental health problem’ ranked high. Specifically, prevalence of 'mental disorders' and 'suicide rate' ranked first, probably because of the rapidly increasing number of mentally ill people and suicides. Also, the ‘Number of mental health professionals’ and ’Mental health expenditures’ were identified as important indices (Table 3). 

**Table 3.** Mental Health Indices

Domain	Index	Ranking
**Mental health status**		
Positive mental health	Subjective health awareness	14
Mental health problems	Drug related deaths	26
	Annual prevalence of mental disorders	1
	Annual prevalence of drug use	9
	Suicide rate (12-month prevalence)	2
	Suicide related deaths (per 100,000 population)	5
	Burden caused by mental disorders (disability-adjusted life years, daly)	8
**Mental health Factor**		
Environmental factors	Social support	10
Personal conditions	Life events (divorce, death, bankruptcy and other depressing events) throughout the year	13
	Life satisfaction	22
	Self-esteem	23
	Flexibility	12
	Limitations in performance due to mental problems	18
**Mental health system**		
Mental health services	No. of psychiatric beds (per 1000 population)	6
	Number of private psychiatric hospitals	17
	Number of general hospitals (with separate psychiatric units)	21
	Long-term hospitalization rate	20
	Use of outpatient services	19
	Self reported use of mental health services	24
	Consumption of psychotropic drugs	16
	Number of disability pensions due to mental disorders	25
	Mean admission days for mental disorders	15
	No. of inpatients at mental health institutions per 100,000 persons	11
	Re-hospitalization rate within 30 days from discharge	27
Human resources	No. of mental health professionals (psychologists, psychiatrists, and other professionals in the field of mental health)per 100,000 population	3
Finance	Mental health expenditures	4
	Mental health per capita expenditures	7

## 5. Discussion

R Despite the fact that in recent decades strong systems of social health indices have been developed, but such indices usually contain only few indices of mental health (13). Wold et al. (2008) identified 35 health index sets. It is noteworthy that many of those index sets contained only few indices related to mental health (19, 20). Recent projects attempting to develop community indices in the European Union, Scotland, Finland and Australia, specifically focused on the mental health and provide a framework and sets of indices (13, 21). Another project has been conducted in Europe in order to establish indices for monitoring the mental health (20, 22, 23). This project has proposed a total of 36 indices for mental health based on the the following categories: 1. Demographic and socio- economic factors, 2. Health status, 3. Determinants of health, 4. Health systems.

There are many social and economic factors, such as the economic crisis, unemployment and poverty in the community which are risk factors for health problems and predispose to mental disorders, suicide and addiction. Mental health systems are faced with many challenges to solve these problems (2, 5). Although the mental health system and community mental health centers in Iran have become more, still more progress is needed to efficiently deal with various mental health problems. So it is necessary to perform research to identify indices mental health in Iran. The set of indices listed in Tables 2 and 3 can be used for setting the roadmap for the development of mental health services in Iran. The final result of the project is the proposal of a set of 27 mental health indices. Similar to other research studies in this field, these indices were selected from the scientific literature and consensus between a sample of experts in the field of mental health (23, 24). This study is a preliminary study for identification of mental health indices that reflects the status of mental health in Iran and provided indices in the areas of mental health status, mental health factors and mental health system. Moreover, the aim of this study was to develop indices of mental health, assessment of the current status of mental health, development of future-oriented mental health programs and monitor the quality of mental health services. Furthermore, this study attempted to present comparable indices by including mental health indices developed by international organizations such as World Health Organization and other also those developed by other countries. The mental health indices presented in this study are expected to be helpful in improving the mental health of the population, evaluation of programs and mental health services, analysis of mental health problems and mental health policies. We hope that the 27 mental health indices explored through the Q-methodology may help both policy makers and mental health professionals. The main strength of this study was the use of Q methodology; because it is based on the perceptions and opinions of individuals and can be used widely in psychology. The major limitation of this study was the fact that the indices developed in this study targeted only adults and not the children, adolescents or elderly people.
